# Three cases of BRAF mutation negative Erdheim-Chester disease with a challenging distinction from IgG4-related disease

**DOI:** 10.1186/s13223-020-00505-2

**Published:** 2021-01-06

**Authors:** David Spoerl, Raphaël André, Aurélie Bornand, Jörg D. Seebach

**Affiliations:** 1 Division of Immunology and Allergy, Department of Medicine, University Hospital, Rue Gabrielle-Perret-Gentil 4, 1205 Geneva, Switzerland; 2 Division of Clinical Pathology, Department of Diagnostics, University Hospital and Medical Faculty, Geneva, Switzerland

**Keywords:** Erdheim-chester disease, Retroperitoneal fibrosis, Renal mass, Histiocytosis, IgG4-related disease

## Abstract

**Background:**

Erdheim-Chester disease (ECD) is a rare non-Langerhans histiocytosis with slow progression over the years that is particularly difficult to diagnose.

**Cases:**

Here we report three cases of ECD without *BRAF* mutation presenting with a renal mass, hairy kidney appearance, and a rather benign course, for which the diagnosis of ECD was delayed, characterized by multiple investigations and unsuccessful treatments attempts. In two cases the distinction from IgG4-related disease required multiple investigations and reevaluation of the clinical, radiological, histological, and immunological characteristics.

**Conclusion:**

A correct diagnosis of ECD may take several years and often requires revisiting previous hypotheses. Reassessment of histological slides and more modern complementary exams such as PET-CT or *BRAF* and *MAPK-ERK* mutation analysis can help to confirm the diagnosis of ECD and to select effective therapy.

To the Editor,

Erdheim-Chester disease (ECD) is a rare non-Langerhans cell histiocytosis, first described in 1930 by William Chester and Jacob Erdheim. It is now considered a histiocytic neoplasm characterized by tissue infiltration of CD68+ CD1a− histiocytes [1]. Here, we report three additional cases of BRAF mutation negative ECD presenting with a renal mass, a rather benign course, for which the diagnosis of ECD was delayed with multiple investigations and unsuccessful treatments attempts.

The first 39-year-old patient with known ulcerative colitis and primary sclerosing cholangitis presented himself in 2013 with macrohematuria and fever. A 3 cm large left superior renal mass was detected on CT, infiltrating the surrounding fat tissue giving a ‘hairy kidney’ appearance, associated with multiple local, mediastinal, axillary and periaortic lymphadenopathies and multiple lytic and partially sclerotic pelvic bone lesions (Fig. 1). In addition, PET-CT showed hypermetabolism of the renal mass and the lymphadenopathies and revealed several bone lesions in the left humeral head and left femoral head. In contrast, bone scintigraphy did not show metabolic activity in the bone lesions. A biopsy of the axillary lymphadenopathy was inconclusive, showing only morphologically normal lymphoid cells and no cell clonality. A second biopsy of the renal mass showed a non-specific chronic inflammatory process. Because of spontaneous resolution of the hematuria and decrease in the size of the tumor mass upon control images no treatment was administered with an uneventful follow-up. In 2016, the renal biopsies were re-evaluated. CD68 and CD163 immunostainings revealed the presence of a considerable amount of histiocytes, negative for anti-CD1a and anti-protein S100 antibodies (Fig. 1), not reminiscent of Langerhans cell histiocytosis. Some of the plasma cells stained positive for IgG but were negative for IgG4. *BRAF* mutations were not detected, neither by immunostaining nor by PCR/sequencing of exons 11 and 15. A final diagnosis of ECD was made. The patient eventually died 1 year later of hepato-biliary carcinoma secondary to primary sclerosing cholangitis. Colon and liver tissue collected in 2007 and 2016, respectively, were re-evaluated *post-mortem*, showing no signs of Langerhans cell histiocytosis and a classical picture of ulcerative colitis.

**Fig. 1:**
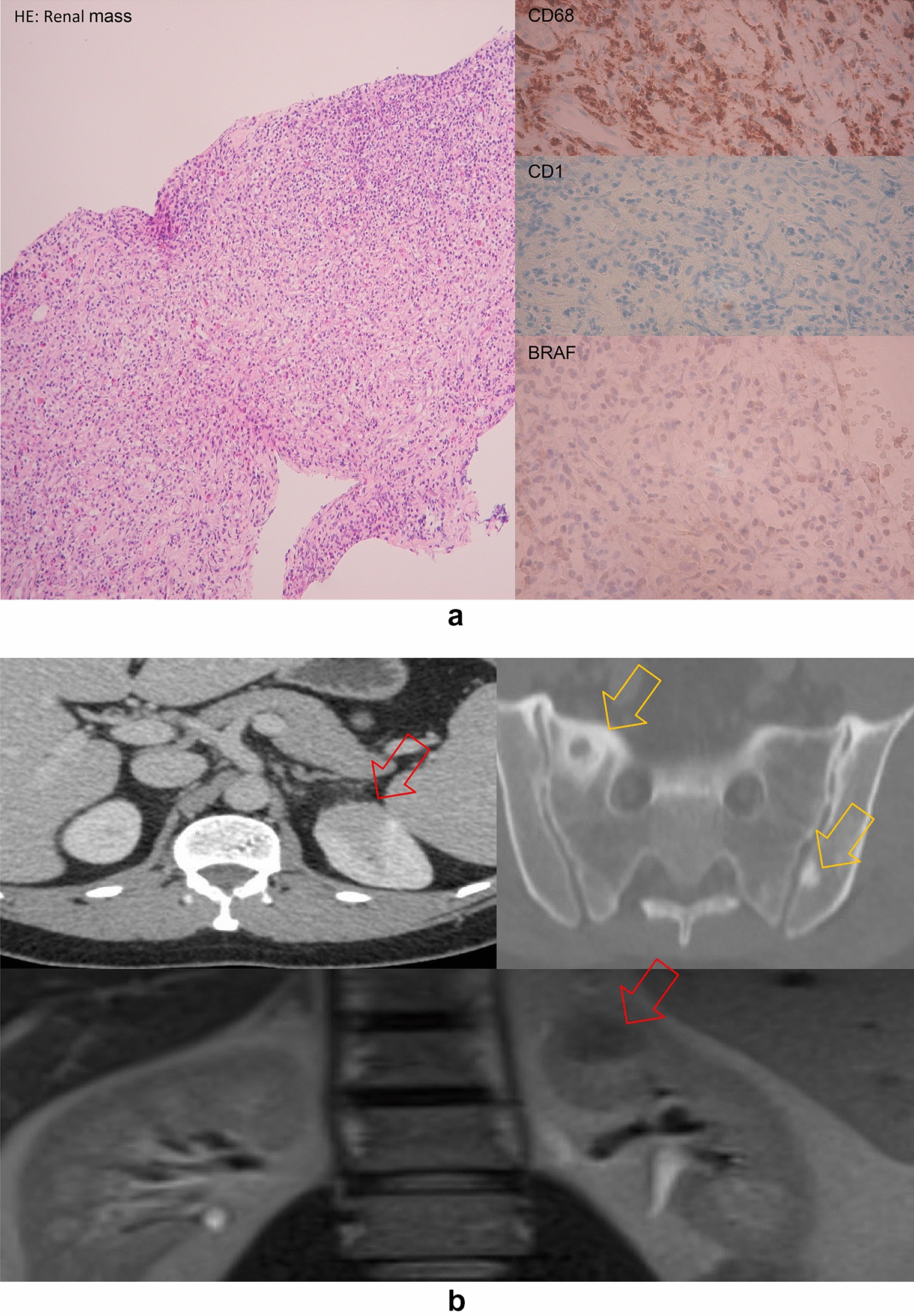
**a** Renal mass histology of case 1: HE and relevant immunohistochemical staining are shown. **b** CT scan and MRI of case 1: Red arrows show the renal mass, yellow arrows point to the osseous lesions

The second 76-year-old patient with a history of hypertension, presented himself in 2005 with diffuse abdominal pain, fever and cough. An angio-MRI showed a soft-tissue mass surrounding the abdominal aorta between the renal and iliac arteries, spreading and infiltrating the perivascular renal tissue. Ureteral medial shift was also visible, but hydronephrosis was not present. Treatment with corticosteroids was introduced for suspected idiopathic retroperitoneal fibrosis (IRF). In 2007, progressive hydronephrosis required ureteral stenting, followed by surgical ureterolysis and bilateral ureteral transposition. Biopsies showed an extensive fibrosis with a mainly collagenous component containing rare fusiform cells corresponding to fibroblasts and a moderate lymphoplasmacytic infiltrate, compatible with IRF. Again, treatment with corticosteroids alone was administered, leading to an improvement but also to adverse side effects including steroid-induced diabetes. In 2013, the patient presented himself with muscular weakness, dysphagia, weight loss, and fever and was referred to our center. A CT scan displayed an extension of the fibrotic process into the mediastinum, enclosing the thoracic aorta and the esophagus. The left kidney displayed signs of atrophy with infiltration of the adipose tissue leading to a ‘hairy kidney’ appearance (Fig. 2). Left nephrectomy was performed and histology showed nephroangiosclerosis, fibroelastosis, interstitial fibrosis of about 40% of the cortical surface, and an prominent inflammatory lymphoplasmacytic infiltrate and macrophage-rich fibrosis surrounding a cyst, with 12 IgG4 positive plasma cells/HPF (diagnostic threshold: > 10 for the kidney) in the setting of concurrent steroid treatment. Storiform diffuse fibrosis and obliterating phlebitis were identified in several areas. Among the infiltrating inflammatory cells, numerous cells showed a large, foamy cytoplasm. These cells stained positive for CD68 and negative for CD1a. The search for *BRAF* mutation was negative by immunostaining and PCR sequencing; bone scintigraphy did not show any lesions. Despite the differential diagnosis of IgG4-related disease, a diagnosis of ECD was made. A treatment with prednisone between 2013 and 2017 resulted in a complete remission. There have been no recurrences since. In 2020 molecular analysis on an Illumina NextSeq using an custom Agilent SureSelect panel of 463 genes, including *BRAF, KRAS, NRAS*, *ARAF, PIK3CA, MAP2K1, ALK* and genes involved *MAPK-ERK* pathway and in other neoplastic disease, did not show any mutations in the previously collected perirenal tissue.

**Fig. 2:**
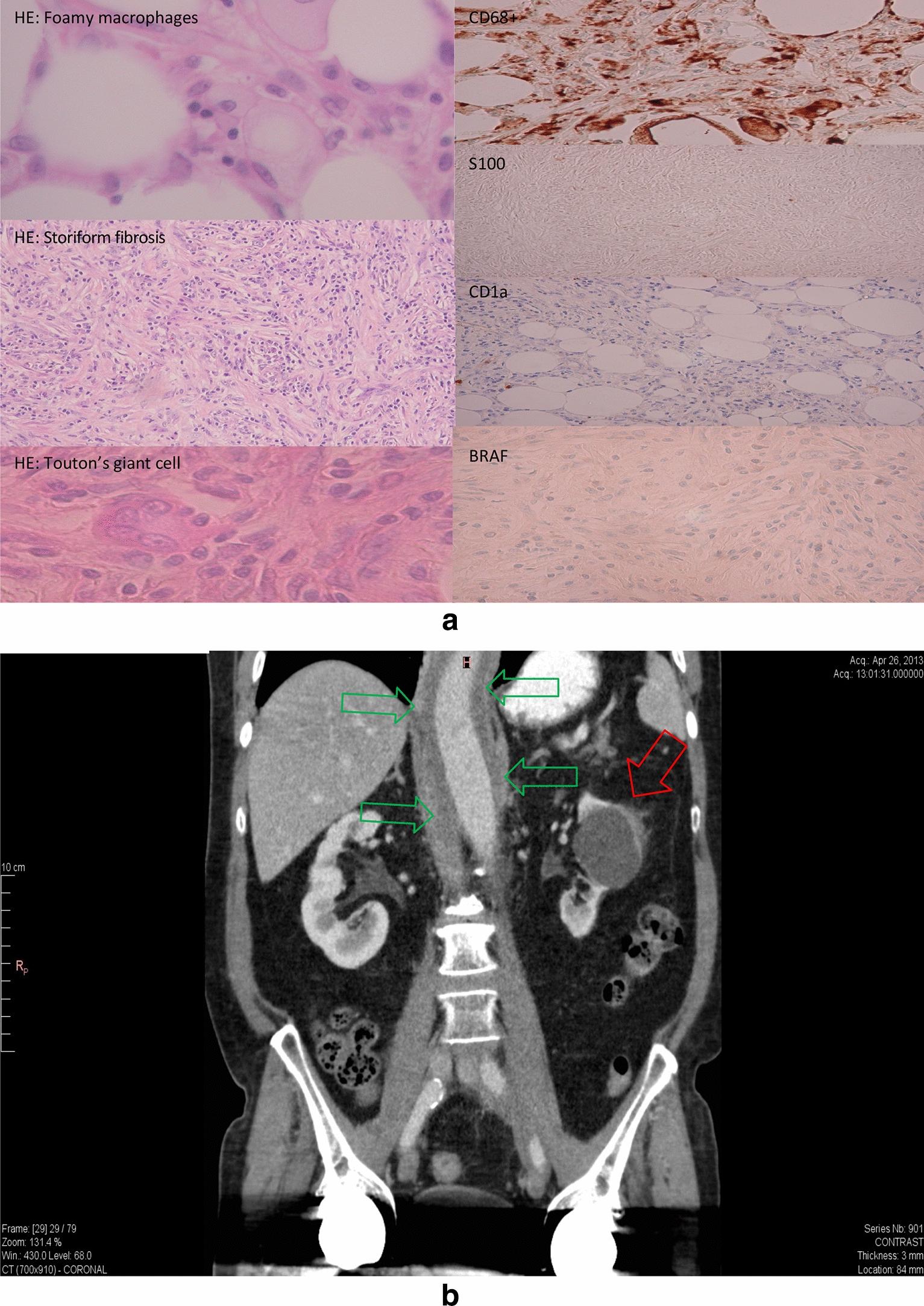
**a** Renal mass histology of case 2: HE and relevant immunohistochemical stainings are shown. **b** CT scan of case 2: Red arrow indicates the renal mass, green arrows the periaortic fibrosis

The third 69-year-old patient underwent abdominal CT scan for gastric pain in 2014 demonstrating a ‘hairy kidney’ peri-renal infiltration (Fig. 3). IgG4 levels were elevated at 2.3 g/l (normal value < 1.35 g/l). A renal biopsy revealed an inflammatory infiltrate constituted of lymphocytes, plasma cells, non-foamy histiocytes and storiform fibrosis. Immunostainings showed macrophages positive for CD68 and CD163, negative for CD1a and protein S100. A rich IgG4-positive plasma cell population was detected (> 10 cells/HPF). IgG4 related-disease was suspected. Treatment with systemic steroids and rituximab was not effective. After 1 year, PET-CT showed new bone lesions (humerus and femurs) and inflammatory markers showed disease progression. These lesions were confirmed by a technetium 99 m bone scintigraphy (Fig. 3). In 2016, femoral bone biopsy showed a hypercellular and trilinear hematopoiesis with interstitial lymphoplasmacytic infiltrate and foamy histiocytes which were positive for CD68 and CD163 but negative for CD1a and protein S100 at immunostainings. Only a minority of plasma cells were positive for IgG4, and *BRAF* staining was negative. No signs of myeloproliferative disease were seen. A diagnosis of ECD was made based on clinical, radiological and histological findings. Interferon-α was discontinued shortly after its introduction due to a flu-like syndrome and mood disorder; systemic corticosteroids and anakinra had no effect on systemic inflammation and fatigue. In 2020, cardiac involvement was found on PET- CT, there was still no sign of myeloproliferative disease, and molecular analysis (as described above for case 2) on renal tissue revealed a DNMT3A mutation of unknown significance with a frequency of 9%.

**Fig. 3:**
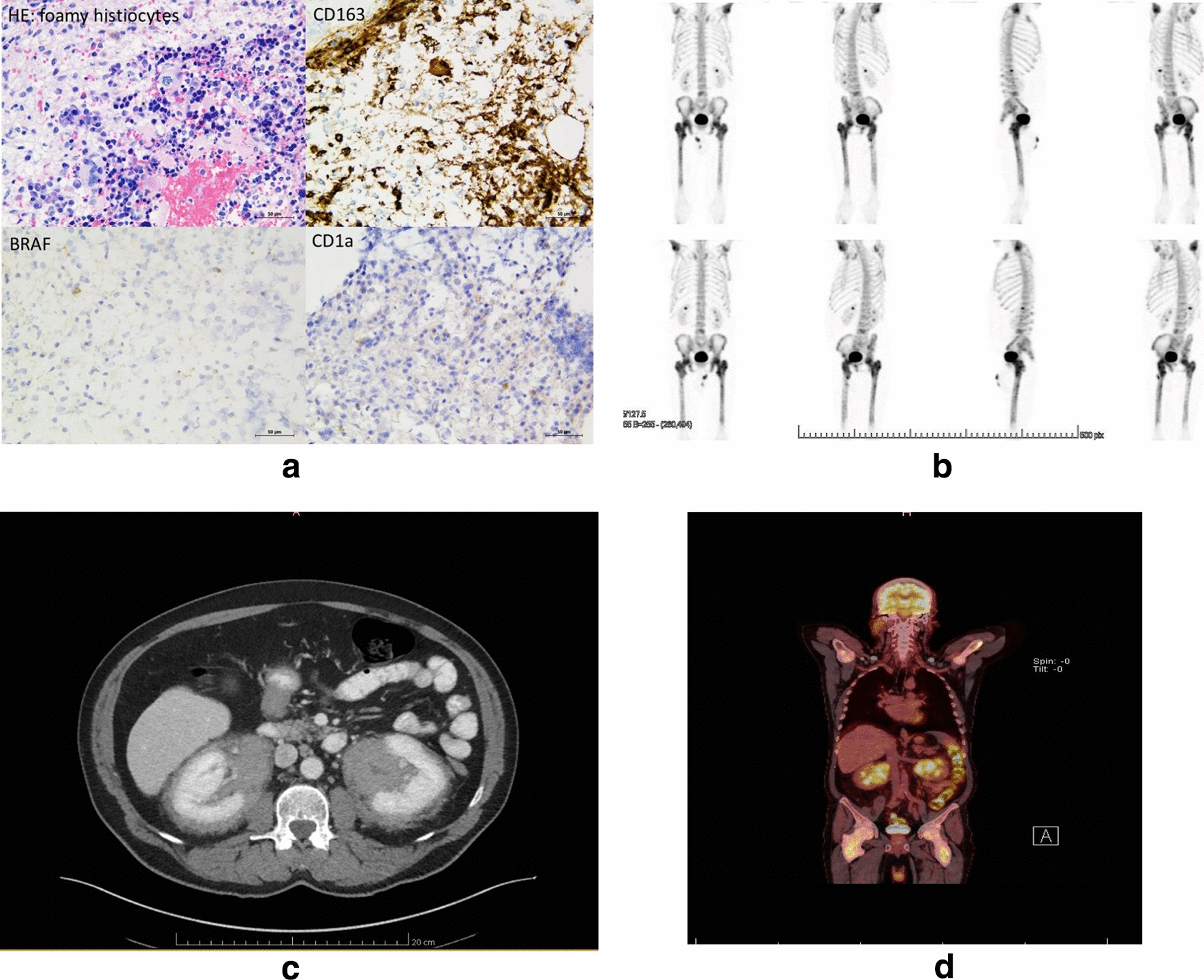
**a** Bone lesion histology of case 3. **b** Technetium-99m bone scintigraphy of case 3. **c** Abdominal CT-scan of case 3 showing peri-renal infiltration without enhancement. **d** PET-scan FDG total body of case 3 showing femoral and humeral hyperintensity

ECD is now considered a neoplasm and the search for the *BRAF* and *MAPK-ERK* pathway mutations is recommended for targeted treatment [1]. *BRAF* mutation is found in 50% of ECD cases [2], but was not present in our patients. These 3 cases illustrate the complexity of diagnosing ECD which caused a significant delay of 9, 8 and 2 years respectively. Involvement of the retroperitoneal space that can mimic IRF was present in all three patients and has been reported in 68% of ECD patients [3]. Skeletal involvement occurs in about 95% of ECD patients [4] and was present in patients 1 and 3. Bilateral cortical sclerosis involving the diametaphyseal regions visible on PET-CT and 99mTc bone scintigraphs are considered virtually pathognomonic of ECD. Around 50–70% of patients with ECD have cardiovascular involvement with periaortic fibrosis (coated aorta), which was present in patient 2, pericardial or myocardial involvement, present in patient 3 [3–5]. The distinction with IgG4-related disease can be challenging due to overlapping clinical manifestations (retroperitoneal infiltration, periaortitis, orbital pseudotumors) and histological similarities [6, 7]. Gianfreda et al. reported elevated IgG4 serum levels in 4/15 ECD patients (26.7%) and two of them also showed IgG4-bearing plasma cells fulfilling the consensus guideline criteria for a histological diagnosis of IgG4-related disease but did not show storiform fibrosis [6]. The overlap between ECD and IgG4-related disease therefore may represent a spectrum, but remains controversial, and the significance of IgG4-bearing plasma cells in ECD needs to be further elucidated. Nevertheless, their presence might indicate a better treatment response to corticosteroids and/or rituximab. In patient 1, IgG4-related disease was initially suspected in 2016 after reappraisal of the CT scan images showing a ‘hairy kidney’ sign and because of the inflammatory infiltration by plasma cells and histiocytes. In patient 2, histology was clearly consistent with IgG4-related disease, however, the presence of foamy macrophages and the characteristic ‘hairy kidneys’ led us to a diagnosis of ECD, without the possibility to firmly exclude IgG4-related disease. Circulating CD19+ CD38+ CD27+ CD20− plasmablasts have been reported to be a hallmark of IgG4-related disease [8]. Patient 1 was not tested before death. Patient 2 had in November 2019 0.28 plasmablasts/μL (within the range reported for healthy controls), while patient had 0.82 plasmablasts/μL (mildly elevated compared to healthy controls) in June 2020. Histology is mandatory for the diagnosis of ECD, but not specific [1]. Indeed, chronic inflammation frequently includes infiltration by CD163+ CD68+ histiocytes, some of which may have a foamy cytoplasm. Thus, diagnosis relies on a combination of clinical, radiological and histological features. Nowadays, ECD can be confirmed in many cases by genetic investigations, which in addition may help to select effective therapy. *BRAF* mutations were not found in any of our 3 patients, moreover case 2 and 3 were tested for and did not show any relevant mutation in the *MAPK-ERK* pathway. Nevertheless, all three patients fulfilled the histological criteria, while only case 3 fulfilled the radiological criteria. Despite the absence of Langerhans cells on histology, a mixed histiocytosis, as seen in some cases of ECD, was not excluded in case 1 who presented with sclerosing cholangitis and ulcerative colitis [9].

The use of interferon α and anakinra as first line treatment in the absence of severe impairment is now proposed [1]. Systemic steroids alone are no longer recommended in the absence of any overall survival benefit. Two of our cases received systemic steroids without benefit whereas patient 2 achieved complete remission with prednisone. For severe disease, targeted therapy (*BRAF* or *MEK* inhibitors) should now be considered.

In conclusion, the correct diagnosis of ECD and distinction from IgG4-related disease requires multiple investigations and reevaluation of the clinical, radiological, biological and immunological characteristics.

## Data Availability

Not applicable.
